# MassCascade: Visual Programming for LC-MS Data Processing in Metabolomics

**DOI:** 10.1002/minf.201400016

**Published:** 2014-04-22

**Authors:** Stephan Beisken, Mark Earll, David Portwood, Mark Seymour, Christoph Steinbeck

**Affiliations:** [a] European Molecular Biology Laboratory – European Bioinformatics Institute (EMBL-EBI) Welcome Trust, Genome Campus, Hinxton, Cambridgeshire, UK; [b] Syngenta Jealott's Hill International Research Centre Bracknell, Berkshire, UK

**Keywords:** Mass spectrometry, Data analysis, Workflow platform, Metabolomics

## Abstract

Liquid chromatography coupled to mass spectrometry (LC-MS) is commonly applied to investigate the small molecule complement of organisms. Several software tools are typically joined in custom pipelines to semi-automatically process and analyse the resulting data. General workflow environments like the Konstanz Information Miner (KNIME) offer the potential of an all-in-one solution to process LC-MS data by allowing easy integration of different tools and scripts. We describe MassCascade and its workflow plug-in for processing LC-MS data. The Java library integrates frequently used algorithms in a modular fashion, thus enabling it to serve as back-end for graphical front-ends. The functions available in MassCascade have been encapsulated in a plug-in for the workflow environment KNIME, allowing combined use with e.g. statistical workflow nodes from other providers and making the tool intuitive to use without knowledge of programming. The design of the software guarantees a high level of modularity where processing functions can be quickly replaced or concatenated. MassCascade is an open-source library for LC-MS data processing in metabolomics. It embraces the concept of visual programming through its KNIME plug-in, simplifying the process of building complex workflows. The library was validated using open data.

## 1 Introduction

Metabolomics studies characterise biological samples and identify metabolites.[1] To investigate the small molecule complement of organisms, liquid chromatography coupled to tandem mass spectrometry (LC-MS^n^) is a routine technique commonly used. LC-MS^n^ is applied in profiling, fingerprinting, or untargeted mode[2] in a variety of areas including environmental,[3] plant[4], and biomedical research[5]. Modern LC-MS^n^ systems can detect more mass traces than ever before thanks to high mass accuracy[6] and resolution[7] producing complex data for every sample.

The data typically consists of mass-to-charge (*m*/*z*), time, and intensity triplets that describe for every detected ion mass the strength of the ion beam and the time it is detected by the spectrometer. Processing and interpreting LC-MS^n^ data is extraordinarily difficult because of the high dynamic range, chemical diversity, and metabolite numbers typically found in metabolome samples. These can exceed 3000 metabolites in eukaryotic cells.[8] The partially convoluted, densely populated signal landscape contains systematic and random noise amongst true signals of varying intensity and shape. Additionally, formation of ion clusters, adducts, or fragmentation implies that many of the extracted peaks or features can belong to the same compound.

Proprietary and free libraries addressing the data processing problem have been developed. These tools can be grouped into three categories: command-line,[9,10] stand-alone graphical user interface (GUI),[11,12] and web-based tools,[13,14] each offering unique advantages, which are reviewed and discussed elsewhere.[15] However, due to the nature of metabolomics data, processing requires complex data analysis workflows, bundling different programs, traversing parameter space, pulling in additional information from databases, and performing statistical multivariate analysis. Consequently, pipelines have been built concatenating existing tools.[16,17]

Workflow platforms such as the Konstanz Information Miner (KNIME)[18] offer the potential for an all-in-one solution. KNIME is a workflow platform that supports a wide range of functionality and has an active bioinformatics community.[19,20] OpenMS,[21] another library for LC/MS data management and analyses has recently been added to its bioinformatics suite. Workflow-based data processing can be described as visual programming. It has the advantage of ease-of-use and rapid development of complex pipelines while maintaining flexibility due to modularity.

Here we present MassCascade and its plug-in MassCascade-KNIME, a library and node-suite for stepwise LC-MS^n^ metabolomics data processing. We give an overview of the architecture of the library and plug-in and discuss the advantages of a unified workflow environment.

## 2 Description

The core library MassCascade and the plug-in MassCascade-KNIME, from here on referred to as ‘plug-in’, are written in Java version 1.7. The open-source projects are released under GPL version 3 and are available on the project hosting website BitBucket, including documentation: https://bitbucket.org/sbeisken/masscascadeknime/wiki/Home

The stand-alone core library contains a collection of data processing algorithms and a visualisation framework, which are wrapped in the plug-in. The plug-in provides its own workflow data model that utilises the core library for data processing and visualisation, hence enabling workflow-based node-wise execution and inspection of data processing tasks. Details about the separate projects can be found below.

### 2.1 Core Library

The core library contains methods for LC-MS^n^ data processing. Each method uses multi-threading. The core instances passed between methods represent essential MS entities. These are defined as follows where *S* (scan) is a set of *m*/*z*-intensity pairs *s* at scan time *t*; *F* (feature or ion trace) is a triplet containing a time vector *t*, an intensity vector *I*, and a characteristic retention time *t_r_* for a given *m*/*z*; *FS* (feature set) is a set of features *F* at an averaged characteristic retention time *t_r_*, i.e. features that are believed – by a chosen criterion such as the inner product between their spectral vectors – to belong together.


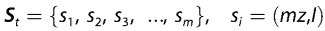



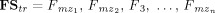


Each method takes a set of parameters including one or many MS instances, applies the method’s function on the instance, and returns a new MS instance. This way a snapshot of the data can be serialised to disk after any processing step if required. That is essential for workflow environments, where intermediate results need to be accessible.

The library supports the HUPO PSI mzML 1.1.0 specification,[22] superseding the older mzXML[23] and mzData[24] formats, and Thermo Scientific’s RAW file format. The methods have been optimised for centroided data. Input data should be centroided with one of the many available file converters such as ProteoWizard[25] or by using the implemented wavelet-based centroider. On read-in, files are individually converted in memory to an internal representation and – optionally – serialized to disk. Smaller files can be quickly processed in a server environment in this way given enough memory.

The library implements methods for noise and signal filtering, as well as methods for feature extraction and deconvolution. Methods for signal identification include database links to PubChem or MassBank, and functions for isotope, adduct, and ion annotation. For a detailed list of implemented algorithms, see section three in the Supplementary Material. The visualisation framework allows data inspection for the core instances. The library works stand-alone enabling its use in compute intensive tasks. For local applications, a plug-in was developed to simplify its use.

### 2.2 KNIME Plug-In

**Figure 1:**
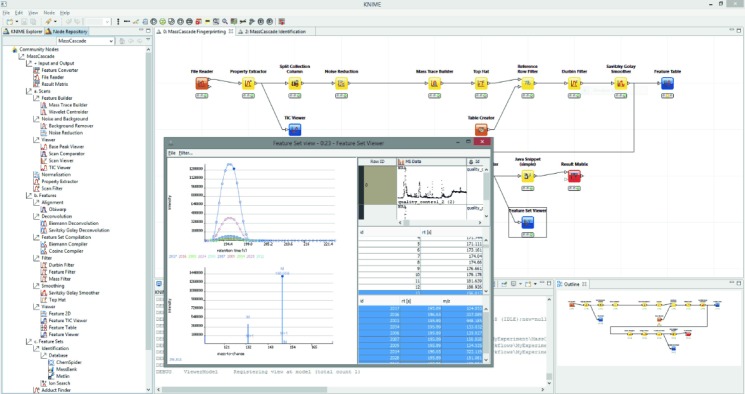
Screenshot of the workflow platform with the MassCascade plug-in loaded. The workflow reads LC-MS samples and carries out several processing steps, represented by nodes. The window in the foreground shows ion chromatograms of features from the same compound spectrum.

The KNIME plug-in encapsulates the methods provided in the MassCascade library and exposes each method to the user as an individual node. The MS instances exchanged between nodes are represented as data cell types that comply with the platform’s tabular data model, hereafter referred to as MS cells. Nodes can be configured through their configuration dialogs, which also validate any set method parameters. After successful execution, the resulting MS cells are accessible via the node’s out-port. Multiple methods can be concatenated to build complex workflows (Figure 1). In- and out-port compatibility is guaranteed by different types of MS cells. Nodes can only be configured for out-ports with MS cells of the same type as required by the in-port.

Nodes for data visualisation are built on top of the core library’s visualisation framework. Every MS cell can be inspected after every execution step, allowing results to be scrutinized and parameters to be adjusted. Different visualisation nodes work only on particular MS cell types. For example, the *Feature Viewer* works only on cells of type *Feature Cell*. Further information about node usage and implementation of additional nodes can be found online in the documentation of the project.

## 3 Results and Discussion

Using the MassCascade-KNIME plug-in, we successfully pre-processed and identified reference samples from 58 compounds – 30 in positive, 16 in negative – and 12 in both ion modes (see section one and two in the Supplementary Material). The files are deposited under “Metabolite Standards for the development and validation of MassCascade”, as part of the data set *MTBLS38* in the MetaboLights database.[26] They, in turn, form part of a metabolomics study on the ripening behaviour of *Solanum lycopersicum* (*MTBLS36*), where MassCascade was used for data processing, analysis, and metabolite identification. In this study, a feature matrix of a total of 219 samples was generated after noise reduction, baseline correction, alignment, and feature annotation to single out significantly different metabolites between the genotypes investigated. In addition, MassCascade enabled rapid fingerprinting of the sample groups to remove outliers, e.g. from erroneous sample preparation, and branching of the processing workflow to explore the data (submission pending). In this application note, we focus on the subset of Metabolite Standards to highlight features of MassCascade in a straight-forward application: identification of reference standards.

Initially, the workflow was built and tested on a single sample to quickly explore the parameter space for every node and find the best set of parameters. Spreadsheets including possible adducts and the reference library were read in for positive and negative ion mode. Once the workflow was set up, all samples were run through the pipeline in batch mode where different ion modes are handled automatically. The main peaks (M+H, M–H), and, if present, isotopes and fragments in MS1 were manually extracted from the result list. To verify identities, the isolated signal and retention time information were compared to a previously established, manually-curated, in-house database. See ‘metabolite_assignment’ in the Supplementary Material for the list of identified metabolites.

We found that MassCascade is useful to automatically narrow down the list of putative signals in the MS samples and identify the main peak plus related peaks in the feature sets. The correct metabolites were identified in all but two samples. In those two samples, the peak shape of the main peak was very narrow (3 scans) and was filtered out. The peaks were recovered by isolating the two samples in the workflow and running a part of it again with less stringent scan width settings.

In contrast to OpenMS, MassCascade provides a higher level interface more accessible to experimentalists. The availability of two (or more) libraries for LC/MS data processing in the same workflow environment gives users a convenient option to harness the advantages of each library, custom tailored to their processing challenges. In comparison, these synergetic benefits can already be seen in the availability and use of multiple cheminformatics toolkits in KNIME.

Building a workflow with the MassCascade plug-in is facilitated by the input-output model of the platform. This form of visual programming reduces the possibility of errors and is particularly user-friendly. The implemented methods can be re-executed and visualised quickly with different parameters, thus, enabling data exploration, supported by generic KNIME nodes and specialized nodes from other providers: Nodes to read comma separated (csv) or Excel (xls) spreadsheets can be used to read lists of contaminants for exclusion. Nodes for row-wise filtering and other table utilities help to organise the data set. The presence of cheminformatics plug-ins in combination with MassCascade opens up additional synergies, e.g. by using managed libraries of small molecules libraries as input for *m*/*z* identification. Another important feature of workflow environments in general is that all workflows, their configuration and even the data, can be exchanged between collaborators, which is extremely important for the expansion and use of metabolomics studies.

## 4 Conclusions

We presented MassCascade, an open-source library for processing LC-MS^n^ metabolomics data, and its plug-in MassCascade-KNIME. The Java library can be used stand-alone or in combination with the plug-in. It comprises basic algorithms for frequent tasks in LC-MS^n^ data processing. Through the plug-in, users can build complex workflows with other KNIME nodes for chem- or bioinformatics or with generic data analysis and visualisation tools, that go beyond the actual MassCascade functionality.

The plug-in offers a modular, step-by-step solution for building complex worfklows with the ability to inspect the output of each method in between nodes. Its ease-of-use and the ready availability of additional nodes with complimentary methods for further data analysis are its key features. Visual workflows also help in the standardisation of data processing and analysis, much needed in the field of metabolomics, where the diversity of instruments and variables is challenging.

Future work involves optimisation of the processing methods in all categories and application of the library and plug-in to complex biological studies as demonstration of the synergistic effects of performing LC-MS^n^ analysis in an open workflow environment.

## Supporting Information

As a service to our authors and readers, this journal provides supporting information supplied by the authors. Such materials are peer reviewed and may be re-organized for online delivery, but are not copy-edited or typeset. Technical support issues arising from supporting information (other than missing files) should be addressed to the authors.

miscellaneous_information
