# Supplementary material for: MassCascade: Visual Programming for LC-MS Data Processing in Metabolomics
Source: Mol Inform. 2014 Apr 22;33(4):307–10. doi: 10.1002/minf.201400016 (PMC4524413; doi:10.1002/minf.201400016)
Supplement: Supplementary file 1 — miscellaneous_information [file minf0033-0307-sd1.pdf]

molecular informatics
models – molecules – systems

Supporting Information

© Copyright Wiley-VCH Verlag GmbH & Co. KGaA, 69451 Weinheim, 2014

1 TESTING

The data was acquired on an Thermo Orbitrap instrument (see '2 Experimental Setup'). The files are deposited under 'Metabolite Standards for the development and validation of MassCascade', as part of the data set `MTBLS38` in the MetaboLights database (www.ebi.ac.uk/metabolights/MTBLS38).

Initially, the workflow was built and tested on a single sample to quickly explore the parameter space for every node and find the best set of parameters. Spreadsheets including possible adducts and the reference library were read in for positive and negative ion mode. Once the workflow was set up, all samples were run through the pipeline in 'batch mode'. The main peaks (`M+H`, `M-H`), and, if present, isotopes and fragments in MS1 were manually extracted from the result list. To verify identities, the isolated signal and retention time information were compared to a previously established, manually-curated, in-house database. The file 'metabolite_assignment.xls' contains the list of identified metabolites.

We found that MassCascade is useful to automatically narrow down the list of putative signals in the MS samples and identify the main peak plus related peaks in the pseudo spectra. The correct metabolites were identified in all but two samples. In those two samples, the peak shape of the main peak was very narrow (3 scans) and was filtered out. The peaks were recovered by isolating the two samples in the workflow and running a part of it again with less stringent scan width settings.

2 EXPERIMENTAL SETUP

Stock solutions of the analytes were prepared in methanol and diluted $1/100$ (V/V) with 50:50 H_2O:MeOH (V/V) to 10 μg/mL.

The platform consisted of a Waters Acquity UPLC system and a Thermo LTQ Velos Orbitrap mass spectrometer operating in the electrospray ionisation mode. The non-targeted LC-MS/MS method used a Waters HSS T3 150 x 2mm, 1.7 μm, Acquity UPLC column.

The solvents used for the assay consisted of 0.2% Formic Acid (Solvent A) and 98/2/0.2 Acetonitrile/Water/Formic Acid (Solvent B). The gradient started at 100% A (hold 2.5 min) at 0.25 mL/min followed by a gentle ramp to 10% B after 7.5 min increasing the flow rate of 0.4 mL/min; then to 100% B after 10 min, hold 2min before equilibrating back to starting conditions after 18 min.

The mass spectrometer was scanned from 85-900 Da (positive ion scans) and 95-900 Da (negative ion scans). Data dependent MS/MS spectra were obtained to aid in the identification of the components detected. The two most intense mass spectral peaks detected in each scan were fragmented to give MS2 spectra. The data was obtained in FT (accurate mass) mode and MS2 obtained in the HCD (higher energy collision dissociation) mode. The LTQ Velos Orbitrap used the Xcalibur control software version 2.1.0.

Non-targeted LC-MS datafiles were converted to mzML using the ProteoWizard program.

3 IMPLEMENTED ALGORITHMS

Tabular description of the main processing algorithms implemented in MassCascade. Utility functions such as filters or web services are not listed. Additional information on the actual usage and a more in-depth description can be found in the help section of the plug-in in KNIME.

Node	Description	Ref.
File Reader	loads mzML and RAW files	—
Mass Trace Builder	sequentially builds ion traces traversing scans	—
Wavelet Centroider	applies a Mexican Hat Wavelet	—
Background Remover	reference background subtraction	[1]
Noise Reduction	random noise reduction	[1]
Obiwarp	Cross-sample feature alignment	[2]
Biemann Deconvolution	feature deconvolution based on a modified Biemann approach	[3]
Savitzky Golay Deconvolution	feature deconvolution based on second derivatives	—
Biemann Compiler	feature set compilation based on a modified Biemann approach	[3]
Cosine Compiler	feature set compilation based on the inner product of pairwise spectral vectors	—
Durbin Filter	feature filter based on a Durbin-Watson statistic	[4]
Savitzky Golay Smoother	feature smoothing based on polynomial fitting	[5]
Top Hat	morphological filter for baseline reduction	[6]
Adduct Finder	combinatorial adduct detection by pairwise m/z comparison	—
Isotope Finder	heuristic isotope finder based on m/z differences	—

[1] P. Zhu, W. Ding, W. Tong, A. Ghosal, K. Alton, S. Chowdhury, Rapid Commun. Mass Spectrom. **2009**, 15631572.
[2] J. T. Prince, E. M. Marcotte, Anal. Chem. **2006**, 78, 614052.
[3] S. E. Stein, J. Am. Soc. Mass Spectrom. **1999**, 10, 770781.
[4] W. Windig, Chemom. Intell. Lab. Syst. **2004**, 77, 206 214.
[5] J. Luo, K. Ying, J. Bai, Signal Processing **2005**, 85, 14291434.
[6] M. van Herk, Pattern Recognit. Lett. **1992**, 13, 517521.
